# Enhanced Resistance to Sliding and Erosion Wear in HVAF-Sprayed WC-Based Cermets Featuring a CoCrNiAlTi Binder

**DOI:** 10.3390/ma19010178

**Published:** 2026-01-03

**Authors:** Lei Zhang, Yue Yu, Xiaoming Chen, Jiaxiang Huo, Kai Zhang, Xin Wei, Zhe Zhang, Xidong Hui

**Affiliations:** 1 Zhejiang-Nepal Small Hydropower Technology Joint Research Center, Standard & Quality Control Research Institute, Ministry of Water Resources, Hangzhou 310012, China; 2 Key Laboratory of Surface Engineering of Equipment for Hydraulic Engineering of Zhejiang Province, Hangzhou Mechanical Design and Research Institute, Ministry of Water Resources, Hangzhou 310012, China; 3 Yellow River Water Resources and Hydropower Development Group Co., Ltd., Zhengzhou 450000, China; 4 State Key Laboratory for Advanced Metals and Materials, University of Science and Technology Beijing, Beijing 100083, China; 5 Water Machinery and Remanufacturing Technology Engineering Laboratory of Zhejiang Province, Hangzhou Jianghe Mechanical and Electrical Equipment Engineering Co., Ltd., Hangzhou 310024, China

**Keywords:** high-velocity air-fuel (HVAF), air-to-fuel ratio (AFR), WC-CoCrNiAlTi, coating, sliding wear, erosion wear

## Abstract

WC-based cermet coatings with a CoCrNiAlTi binder were fabricated on 04Cr13Ni5Mo stainless steel substrates using the atmospheric high-velocity air–fuel (HVAF) spraying process. The influence of the air-to-fuel ratio (AFR) on the microstructure, mechanical properties, and wear resistance of the WC-CoCrNiAlTi coatings was systematically investigated. The results indicate that the WC-CoCrNiAlTi coatings primarily consisted of WC, (Co, Ni)_3_W_3_C and a face-centered cubic (FCC) binder phase. As the AFR increased, the formation of the (Co, Ni)_3_W_3_C phase gradually decreased. Concurrently, the coating density improved, which was attributed to the enhanced particle melting state and increased flight velocity, leading to better flattening upon impact. The average microhardness of the WC-CoCrNiAlTi coatings gradually increased with an increasing AFR. The coating produced at an AFR of 1.130 exhibited the highest microhardness of 1355.68 HV_0.2_. Both the friction coefficient and the wear rate of the coatings decreased progressively as the AFR increased. At the optimal AFR of 1.130, the coating demonstrated the lowest friction coefficient (0.6435) and wear rate (1.15 × 10^−6^ mm^3^·N^−1^·m^−1^), indicating a wear resistance 34.85 times that of the stainless steel substrate. Furthermore, the slurry erosion weight loss rate of the WC-CoCrNiAlTi coatings decreased gradually with increasing AFR. The coating sprayed at an AFR of 1.130 showed the minimum erosion rate (1.70 × 10^−6^ g·cm^−2^·min^−1^), which was 24.04 times lower than that of the substrate. The erosion mechanism of the WC-CoCrNiAlTi coatings was identified as the fatigue-induced removal of WC particles under alternating stress. The ductile high-entropy alloy (HEA) binder effectively protects the brittle WC phase through adaptive deformation, thereby significantly mitigating coating damage.

## 1. Introduction

In hydraulic and hydropower engineering, turbine overflow components are subjected to severe erosion by high-speed sediment-laden water flow over long periods, leading to material failure and degradation of equipment performance, which seriously threatens the operational safety and economy of hydraulic facilities [1]. To enhance the service life of key components in harsh abrasive environments, thermal-sprayed cermet coating technology has been widely adopted [2]. By depositing composite materials with high hardness and wear resistance onto substrate surfaces, this technique effectively mitigates sediment erosion and cavitation damage [3]. Currently, coating systems such as WC-10Co4Cr and Cr_3_C_2_–NiCr, which are commonly used in hydraulic engineering, heavily rely on cobalt as the core element of the binder phase [4]. Although cobalt provides good wettability and toughness, it has several limitations and shortcomings as a binder in cermets: First, the wear resistance of the WC-Co cermet is highly dependent on the state and hardness of the binder. Studies have shown that during service, the cobalt binder wears away prior to the WC phase, leading to the premature loss of carbide support and reduced overall wear resistance [5]. Therefore, balancing adequate toughness with maximized hardness remains a critical challenge for WC-based cermets. Additionally, compared to the carbide, the cobalt binder exhibits inferior chemical stability, making it prone to preferential corrosion and oxidation [6], which significantly compromises the overall performance of the cermet. Thus, the corrosion resistance of traditional cobalt-bonded carbides needs improvement, especially in corrosive environments such as hydropower projects. Economic factors are also non-negligible. With the growing demand for cobalt resources in the global new energy vehicle industry, some countries have classified cobalt as a scarce resource, leading to a sharp increase in market prices [7]. Consequently, the manufacturing cost of cobalt-bonded cermets continues to rise. Furthermore, the carcinogenic potential of cobalt has attracted widespread attention, with accumulating evidence indicating that long-term inhalation of cobalt compounds can lead to respiratory tumors. Thus, developing low-cobalt or cobalt-free, high-performance novel binder materials has become an important research direction in the field of wear-resistant coatings [8].

In recent years, high-entropy alloys have attracted widespread attention due to their unique multi-principal element design concept and excellent comprehensive properties. These alloys typically consist of four or more principal elements in near-equimolar ratios and exhibit high-entropy effects, lattice distortion effects, and sluggish diffusion effects [9], resulting in outstanding combinations of strength and toughness, corrosion resistance, and wear resistance. Among various HEA systems, CoNiCr-based alloys and their derivatives, which typically form an FCC structure, demonstrate excellent plasticity and environmental stability [10]. They are considered highly promising as a new-generation cermet binder to replace traditional CoCr alloys [11,12]. This offers a new pathway for designing toughened coatings suitable for hydraulic conditions. On the other hand, the selection of thermal spraying technology plays a decisive role in the final performance of cermet coatings. Compared with conventional high-velocity oxygen-fuel (HVOF) spraying, the HVAF technique uses air for combustion, resulting in a lower flame temperature but higher particle acceleration capability [13]. This significantly suppresses the decomposition and decarburization of carbides such as WC during flight, reduces the formation of brittle phases, and improves coating density and bonding strength [14,15]. HVAF is particularly suitable for preparing thermally sensitive materials and multi-phase heterogeneous coatings, and has recently demonstrated significant advantages in high-end equipment remanufacturing and protection. The EU-funded CoBRAIN project utilizes HVAF technology—known for its versatility and low environmental impact—to produce hard metal coatings free of toxic and critical materials, serving as an alternative to traditional tungsten carbide–cobalt (WC-Co) and hexavalent chromium-based coatings [16]. However, research on the preparation of WC-based composite coatings with HEA binders via HVAF and systematic investigation of their microstructure evolution and wear-erosion mechanisms remains scarce.

Based on this, this study innovatively proposes a low-cobalt non-equiatomic CoCrNiAlTi HEA as the binder for WC ceramic particles, aiming to replace traditional high-cobalt cermets. In this HEA system, Co, Cr, and Ni primarily ensure a stable FCC structure. A certain amount of Co enhances the wettability between WC and the binder, improving interfacial bonding and fracture toughness. Cr element promotes the formation of a dense passivation film on the surface, thereby enhancing corrosion resistance. Ni element helps maintain the toughness of the matrix. The addition of Al and Ti elements can induce solid solution strengthening and the formation of ordered phases, which are expected to improve microstructural stability and oxidation resistance. WC-CoCrNiAlTi cermet coatings were prepared on 04Cr13Ni5Mo stainless steel substrates by HVAF. This HEA binder design not only reduces cobalt content but also achieves a toughness–strength balance through multi-component synergistic effects. The influence of the key process parameter—AFR—on the phase composition, microstructure, mechanical properties, and slurry erosion resistance of the coatings was systematically investigated. The relationship between particle melting state, flattening behavior, and coating performance was revealed, and mechanisms of fatigue spalling and adaptive deformation under sediment wear conditions were elucidated. This study aims to provide a theoretical basis and technical pathway for the development of a new generation of cost-effective anti-abrasion coatings, and has significant implications for promoting the application of HEAs in the field of engineering protection.

## 2. Experimental Section

### 2.1. Preparation of Coatings

The WC-CoCrNiAlTi coating was fabricated using elemental metal powders for the high-entropy alloy (HEA) binder phase and carbide (WC) powders. The HEA raw elemental metal powders include cobalt (Co), chromium (Cr), nickel (Ni), aluminum (Al), and titanium (Ti) powders (Zhongnuo, Beijing, China), all with purities higher than 99.9% and particle sizes ranging from 15 to 45 μm. The WC powder consisted of 60% micron-sized and 40% nano-sized WC particles (Yijin, Beijing, China). The weight ratio of the WC powder to the HEA raw elemental metal powders is 5.5:1. According to the stoichiometric composition Co_60_Cr_25_Ni_5_Al_5_Ti_5_, elemental metal powders were weighed and loaded into a stainless-steel jar. Ball milling was then carried out in a planetary ball mill (QM-3SP04, Nanda, Nanjing, China) for 20 h with a ball-to-powder weight ratio of 8:1 and a rotational speed of 600 r/min. Subsequently, WC powder was added and mixed in a drum mixer (XQM-6, Tianchang, Changsha, China) for 6 h using a ball-to-powder weight ratio of 1:1 and a rotational speed of 120 r/min. Martensitic stainless steel 04Cr13Ni5Mo was selected as the substrate. Prior to spraying, the substrates were ultrasonically cleaned with alcohol and roughened by alumina grit blasting, resulting in a surface roughness (Ra) of approximately 7.0 μm. The WC-CoCrNiAlTi coatings were deposited using an HVAF spraying system (AcuKote, Kermetico, Benicia, CA, USA) equipped with an AK-06 gun. A robot (MOTOMAN ES165D, Yaskawa, Fukuoka, Japan) was employed to control the spray traverse velocity and uniformity. Compressed air was used to cool the substrate and coating during spraying to prevent overheating. The process parameters were as follows: air pressure 0.624 MPa; propane pressures of 0.552 MPa, 0.565 MPa, and 0.579 MPa; powder feed rate 3.5 r/min; torch traverse speed 1000 mm/s; spray distance 200 mm. The coating thickness was controlled at approximately 300 μm. The corresponding air-to-fuel pressure ratios (AFR) under these parameters were 1.130, 1.104, and 1.078, and the resulting WC-CoCrNiAlTi coating samples were designated as AFR-1.130, AFR-1.104, and AFR-1.078, respectively.

### 2.2. Microstructure Characterization

The phase composition of the coatings was characterized by X-ray diffraction (XRD, X’Pert PRO, Panalytical B.V., Almelo, The Netherlands) using a Cu Kα radiation source operated at 40 kV and 40 mA. Scans were performed over a 2θ range of 5° to 95° with a step size of 0.02° and a scanning speed of 0.04°/s. Prior to testing, the coating surfaces were ground polished sequentially with 180-, 600-, 1200-, and 2400-grit SiC abrasive papers and then with 9, 3, and 0.5 μm diamond suspensions to achieve a smooth surface with a roughness (Ra) of about 0.5 μm. The cross-sectional microstructure of the coatings was examined using a field emission scanning electron microscope (SEM, SUPRA55, ZEISS, Oberkochen, Germany) at an accelerating voltage of 15 kV. The local chemical composition and elemental distribution were analyzed using an energy-dispersive X-ray spectrometer (EDS, X-MaxN20, Oxford, UK) attached to the SEM. The wear scar morphology was also observed by SEM, and the chemical composition of the worn surfaces was analyzed by EDS.

### 2.3. Micromechanical Properties

The microhardness distribution across the coating cross-section was measured using a Vickers hardness tester (HXD-1000TMC, Taiming, Shanghai, China) under a load of 1.96 N applied for 10 s.

### 2.4. Sliding Wear Testing

Dry sliding reciprocating wear tests were conducted at room temperature using a tribometer (UMT Tribo Lab™, BRUKER Corporation, Ettlingen, Germany). A Si_3_N_4_ ceramic ball with a diameter of 6.35 mm was used as the counterface. The test parameters were: load 30 N, frequency 5 Hz, stroke length 10 mm, and test duration 120 min. Prior to testing, the coating surfaces were ground sequentially with SiC abrasive papers and diamond suspension to achieve a smooth surface with a roughness (Ra) of about 0.5 μm. The three-dimensional morphology of the wear scars was characterized using a laser confocal microscope (Up-Lambda 2, Rtec Instruments, San Jose, CA, USA). The wear volume was calculated using Gwyddion v2.52 image analysis software. The volumetric wear rate (*R*_v_, mm^3^/(N·m)) of the samples was calculated using the following equation:

(1) $$R_{v}=∆V/(F\cdot L)$$

where Δ*V* is the wear volume (mm^3^), *F* is the normal load (N), and *L* is the total sliding distance (m).

### 2.5. Erosion Wear Testing

Slurry erosion wear tests were performed on the coatings using an slurry erosion wear tester (LMT-200, Zhongkekaihua, Lanzhou, China). The slurry was prepared with a quartz sand-to-water mass ratio of 2:3. Coating samples with dimensions of 20 mm × 20 mm × 5 mm were mounted on a rotating blade holder with the erosion surface perpendicular (90°) to the rotation direction. The linear rotation speed was 35 m/s. The test duration was 54.5 h, with samples extracted periodically for weighing during the test. Prior to testing, the coating surfaces were ground sequentially with SiC abrasive papers and diamond suspension to achieve a smooth surface with a roughness (Ra) of about 0.5 μm. After drying, the samples were weighed using an electronic analytical balance (LE225D, Sartorius AG, Göttingen, Germany) with an accuracy of 0.1 mg. The slurry erosion weight loss (*W*_e_, g/cm^2^) and erosion rate (*R*_e_, g/(min·cm^2^)) were calculated using Equations (2) and (3), respectively:

(2) $$W_{e}=(M_{0}-M_{f})/S$$

(3) $$R_{e}=(M_{0}-M_{f})/(S\cdot t)$$

where *M*_0_ is the initial weight (g), *M*_f_ is the weight after erosion for time t (g), *S* is the erosion surface area (cm^2^), and *t* is the erosion time (min). After testing, the micromorphology of the eroded coating surfaces was observed by SEM.

## 3. Results and Discussion

### 3.1. Microstructure

**Figure 1:**
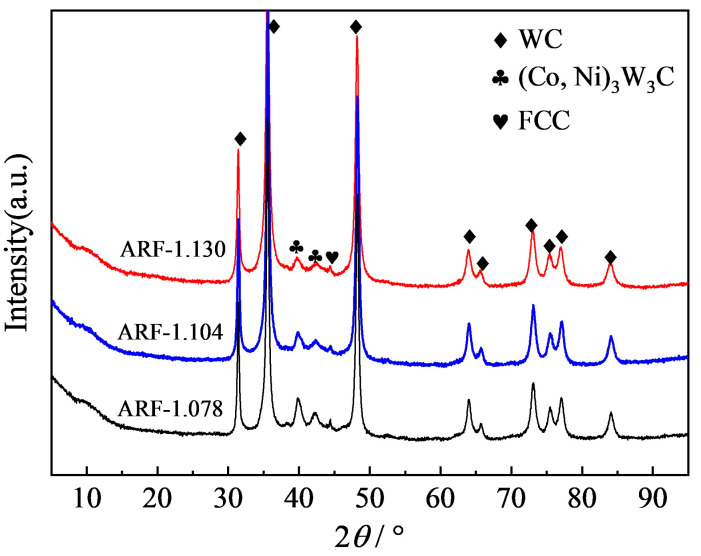
XRD patterns of WC-CoCrNiAlTi cermet coatings.

Figure 1 shows the XRD patterns of WC-CoCrNiAlTi coatings fabricated at different AFRs. It can be observed that all coatings are primarily composed of WC hard phase, (Co, Ni)_3_W_3_C complex carbides, and an alloy binder phase with an FCC structure. The formation of the FCC binder phase can be attributed to two key processes: first, during the high-energy ball milling stage, the elemental metal powders undergo preliminary alloying via mechanical alloying; second, during the HVAF spraying process, the semi-molten state of the powder and high-velocity impact further promote inter-element interdiffusion and alloying, ultimately leading to the formation of the FCC-structured binder phase. The formation of the (Co, Ni)_3_W_3_C phase is mainly attributed to the interdiffusion reaction between W and C atoms—released from the partial decomposition of WC particles during thermal spraying—and elements such as Co and Ni in the binder phase [17]. This phase is structurally similar to the Co_3_W_3_C phase commonly found in conventional WC-CoCr-based coatings. However, due to the partial substitution of Co by Ni, a multicomponent (Co, Ni)_3_W_3_C carbide is formed, reflecting the characteristic phase composition of the high-entropy binder. With increasing AFR, the intensity of the characteristic diffraction peaks of the (Co, Ni)_3_W_3_C phase—particularly at around 2θ = 41.9° and 42.5°—significantly decreases, indicating a gradual reduction in the formation of this phase. This phenomenon can be attributed to changes in the thermal and kinetic behavior induced by an increased AFR: In HVAF processes, where air is used as the combustion gas, the flame temperature (approximately 1960–2010 °C) is significantly lower than that of oxygen-fueled processes such as HVOF. This inherently helps suppress the decomposition and oxidation of WC. However, an excessively low AFR (fuel-rich condition) can lead to a relatively higher flame temperature and overheating of the powder particles, thereby increasing the opportunity for reactions between WC and oxygen. In contrast, a higher AFR is typically accompanied by an increased gas flow velocity, which enhances the flight speed of the powder particles [18]. This reduces the residence time of the particles in the high-temperature flame, shortens their thermal exposure, and effectively mitigates the degree of WC decomposition as well as the diffusion of W and C atoms into the binder phase. As a result, the formation of secondary (Co, Ni)_3_W_3_C carbides is effectively suppressed. These results demonstrate that the phase composition of the coating can be controlled to some extent by adjusting HVAF process parameters, thereby reducing the formation of brittle phases.

**Figure 2:**
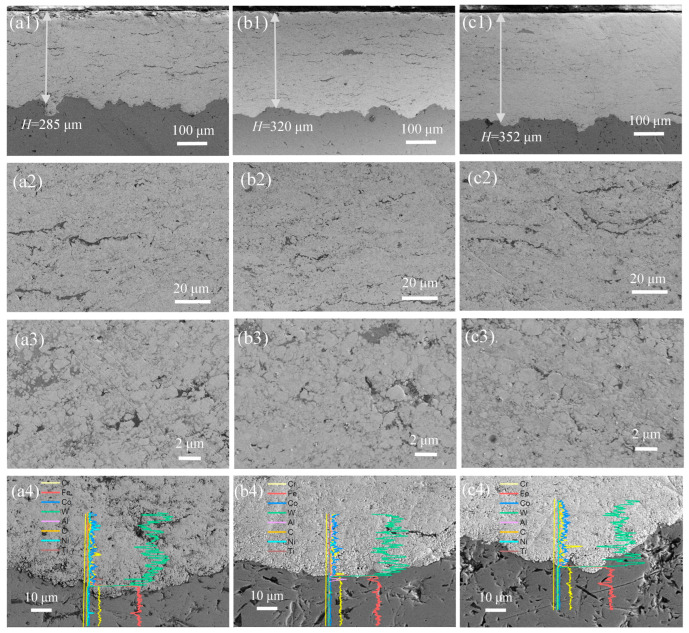
The cross-section morphologies of WC-CoCrNiAlTi cermet coatings for AFR-1.078 (**a1**–**a4**), AFR-1.104 (**b1**–**b4**) and AFR-1.130 (**c1**–**c4**): (**a1**–**c1**) Overall morphologies; (**a2**–**c2**) Low-magnification cross-section image; (**a3**–**c3**) High-magnification cross-section image; (**a4**–**c4**) EDS line-scanning profiles across the interface.

Figure 2(a1–c1) presents the cross-sectional microstructures of the WC-CoCrNiAlTi coatings. All coatings exhibit a typical lamellar stacking morphology. A mechanical bonding interface is formed between the coating and the substrate without visible gaps, indicating good adhesion. As shown in Figure 2(a2), under a lower AFR (1.078), the coating exhibits higher porosity and lower density. Under higher magnification, the light WC phase and the dark HEA binder can be clearly distinguished based on contrast differences. The coating consists of hard phase particles ranging from micron to nano scales embedded in a metallic binder. This multi-scale distribution of the hard phase contributes synergistically to enhancing the hardness of the coating. As the AFR increases, defects such as pores are significantly reduced, and the density of the coating markedly improves, as illustrated in Figure 2(a3–c3). Under high-AFR conditions, no significant structural defects are observed except for minor localized gaps. Moreover, an increase in coating thickness is noted with rising AFR. The increased AFR enhances the velocity of the flame stream and in-flight particles, thereby improving the flattening degree of molten and semi-molten particles upon impact. Under the combined effects of impact pressure and capillary forces, the binder undergoes plastic flow and migrates into the interstices between WC particles, progressively filling or reducing pores [19]. Consequently, a suitably elevated AFR promotes inter-particle bonding and enhances coating density. Simultaneously, the improved adhesion between particles facilitates the deposition process and contributes to an increase in the final coating thickness. Based on the high-magnification SEM images and EDS line-scanning profiles of the coating/substrate interface (Figure 2(a4–c4)), it can be observed that all interfaces of coatings at different AFRs are in a state of mechanical interlocking, with a distinct interface between them. The EDS line scan shows an abrupt change in all elemental signals at the interface, with no gradient transition zone observed, indicating no elemental interdiffusion across the interface. These features indicate that the bonding between the HVAF-sprayed coating and the substrate relies on mechanical interlocking rather than metallurgical bonding.

**Figure 3:**
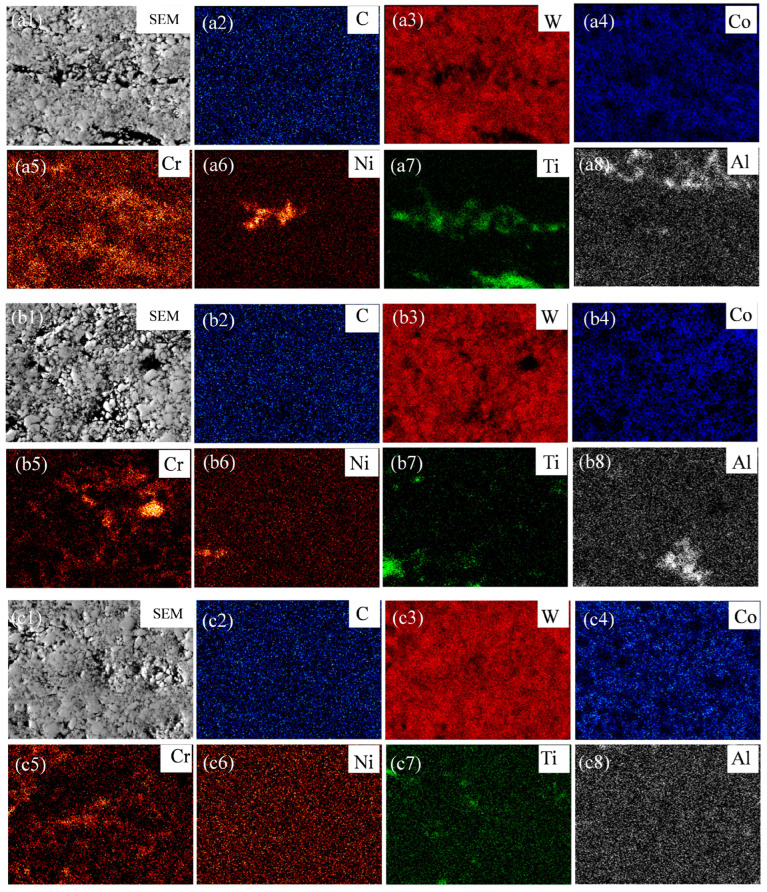
SEM-EDS results of the HVAF-sprayed WC-CoCrNiAlTi coating for AFR-1.078 (**a1**–**a8**), AFR-1.104 (**b1**–**b8**) and AFR-1.130 (**c1**–**c8**): (**a1**–**c1**) SEM images; (**a2**–**c2**) C-mappings; (**a3**–**c3**) W-mappings; (**a4**–**c4**) Co-mappings; (**a5**–**c5**) Cr-mappings; (**a6**–**c6**) Ni-mappings; (**a7**–**c7**) Ti-mappings; (**a8**–**c8**) Al-mappings.

Further SEM-EDS elemental analysis was conducted on the coating, with the results shown in Figure 3. The elemental mappings indicate that C is distributed to some extent in both the WC regions and the binder, suggesting relatively uniform distribution within the coating. W is enriched in the WC hard phase regions, and an amount of diffusely distributed W is also detected in the binder phase. These results confirm that partial decomposition of WC occurs during thermal spraying [20], allowing W and C atoms to diffuse into the binder region and react preferentially with elements such as Co and Ni in the CoCrNiAlTi HEA, forming carbide phases such as (Co, Ni)_3_W_3_C. At a lower AFR (AFR-1.078), localized enrichment of certain elements is observed, as shown in the elemental maps, particularly Cr, Ni, and Ti in the binder phase. As the AFR increases, the uniformity of elemental distribution improves significantly. The EDS mapping images revealed that at a higher AFR (AFR-1.130), the signal distribution of elements such as Cr, Ni, and Ti within the binder phase region became more uniform. The presence of local bright or dark spots—indicative of segregation—was reduced, qualitatively suggesting improved elemental homogeneity. This is mainly due to the more sufficient oxygen supply at higher AFRs, which promotes better melting of the sprayed particles and facilitates elemental diffusion and homogenization.

Table 1 shows the chemical composition of different regions in the WC-CoCrNiAlTi coating under various Air-to-Fuel Ratios (AFR). It is evident that within the WC particle regions, the overall composition remains stable, with no significant diffusion of binder phase elements observed. However, W was detected in the binder phase regions, originating from the dissolution of WC under high-temperature conditions and the subsequent diffusion of W. Furthermore, the measured contents of Al and Ti in the binder phase of all coatings were lower than their nominal values. This discrepancy can be attributed not only to the dilution effect by W element but also potentially to the preferential volatilization or oxidation of elements such as Al and Ti during the spraying process [21,22]. As the AFR increases, the W content in the binder phase shows a decreasing trend. This is due to the reduced flame temperature and shortened thermal exposure time, which thereby effectively suppresses the decomposition of WC, decreases the diffusion of W into the binder phase. In summary, the dissolution of WC during thermal spraying and its subsequent reaction with the HEA binder to form (Co, Ni)_3_W_3_C can be explained by the following three-step process [17]:

2WC → W_2_C + C (4)

W_2_C → 2W + C (5)

WC + 2W + 3M → M_3_W_3_C (6)

It is noteworthy that an excessively high AFR, which implies a more abundant oxygen supply, can conversely exacerbate the oxidation and decarburization of WC particles. Thus, AFR is a core HVAF process parameter that requires precise control and is critical for obtaining WC- CoCrNiAlTi coatings with high performance, low decarburization, and enhanced durability.

### 3.2. Microhardness

**Figure 4:**
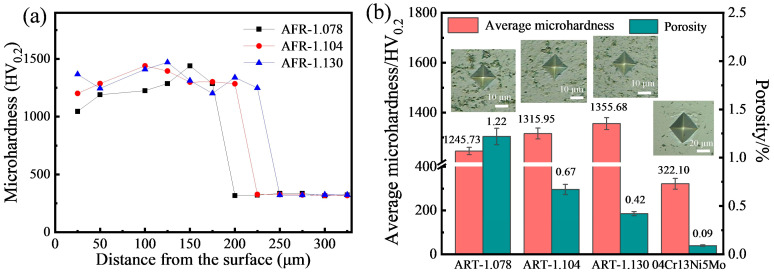
Microhardness and porosity of HVAF-sprayed WC-CoCrNiAlTi coatings: (**a**) microhardness distribution; (**b**) average microhardness and porosity values.

Figure 4a displays the microhardness distribution curves along the cross-section of the WC-CoCrNiAlTi coatings. It can be observed that the microhardness values of all samples remain relatively stable within the coating region but decrease significantly near the coating/substrate interface, gradually approaching the substrate hardness (approximately 320 HV_0.2_). As shown in Figure 4b, the average microhardness of the coatings gradually increases with increasing AFR. The maximum microhardness of 1355.68 HV_0.2_ is achieved at an AFR of 1.130. This hardness is significantly higher than that of some reported thermally sprayed WC cermet coatings, including HVOF-sprayed WC-CoCr coatings (1059.09 –1182.07 HV) [23] and HVAF-sprayed WC-NiCr coatings (870 ± 243 HV_0.3_) [24], and is comparable to or slightly higher than that of HVAF-sprayed WC-CoCr coatings (1260 ± 40 HV_0.3_) [15].

The variation in coating hardness is closely associated with its phase composition and microstructure, resulting from the combined effect of phase composition and microstructural defects (particularly porosity). On one hand, under low-AFR conditions, the hard and brittle decomposition phases (such as (Co, Ni)_3_W_3_C) formed in the coating possess high intrinsic hardness [20], but the overall loose structure of the coating limits the improvement in macroscopic hardness. On the other hand, the density of the coating plays a crucial role in determining its hardness. As evident from the indentation morphologies of different coatings in Figure 4b, the sample prepared at AFR-1.078 exhibits numerous pores around the indentations, indicating insufficient melting of particles under these conditions. This results in poor inter-particle bonding and inadequate internal compressive stress within the coating, leading to lower hardness values [25]. Furthermore, regions near pores are prone to collapse during indentation, which can affect the accuracy of the hardness measurements and introduce deviations in the statistically averaged hardness values. As shown in Figure 4b, the porosity of the coating gradually decreases with the increase in AFR. As discussed in Section 3.1, this is attributed to high-velocity impact promoting particle flattening and spreading, thereby reducing incomplete filling and pore formation. As a result, the denser microstructure contributes to higher microhardness, as exemplified by the AFR-1.130 coating, where significantly reduced porosity outweighs the effect of having fewer hard/brittle phases, leading to higher microhardness.

### 3.3. Sliding Wear Performance

**Figure 5:**
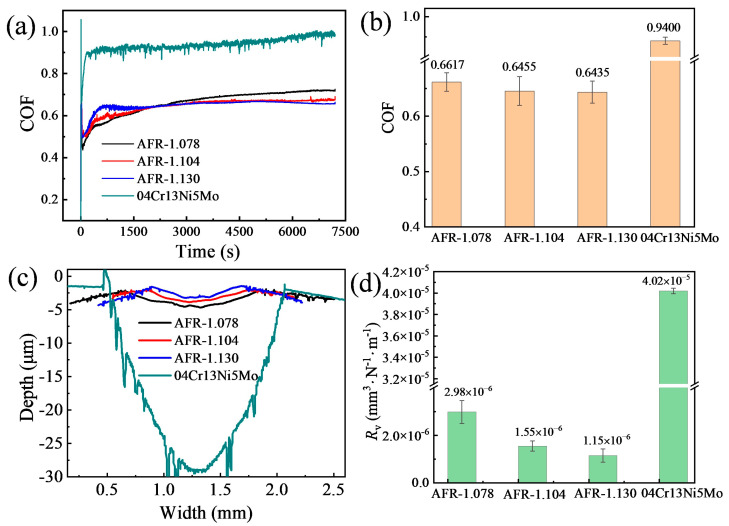
Friction and wear properties of HVAF-sprayed WC-CoCrNiAlTi coatings: (**a**) friction coefficient curves; (**b**) average friction coefficient; (**c**) cross-sectional profiles of wear scars; (**d**) wear rate.

Figure 5a shows the variation in the friction coefficient with time for the WC-CoCrNiAlTi coatings and the substrate under dry sliding conditions. It can be observed that the friction coefficients of all coatings gradually increase during the initial stage and stabilize after running-in periods, entering a steady-state friction stage. As seen in Figure 5b, the average friction coefficient of the coatings gradually decreases with increasing AFR. The AFR-1.130 coating exhibits the lowest friction coefficient of 0.6435. This phenomenon is closely related to the microstructure and phase composition of the coatings. Combined with the XRD results in Figure 1, it is evident that the formation of the (Co, Ni)_3_W_3_C phase, formed by the decomposition of WC, decreases with increasing AFR. Under low-AFR conditions, the coating contains more hard and brittle phases, which are prone to brittle spalling during friction, generating abundant wear debris and exacerbating abrasive wear [26]. This leads to significant fluctuation in the friction coefficient and an elevated average value. In contrast, the coating prepared at a high AFR exhibits a more uniform phase distribution, a relatively smooth wear surface, and less wear debris, resulting in a more stable friction process and a lower friction coefficient.

**Figure 6:**
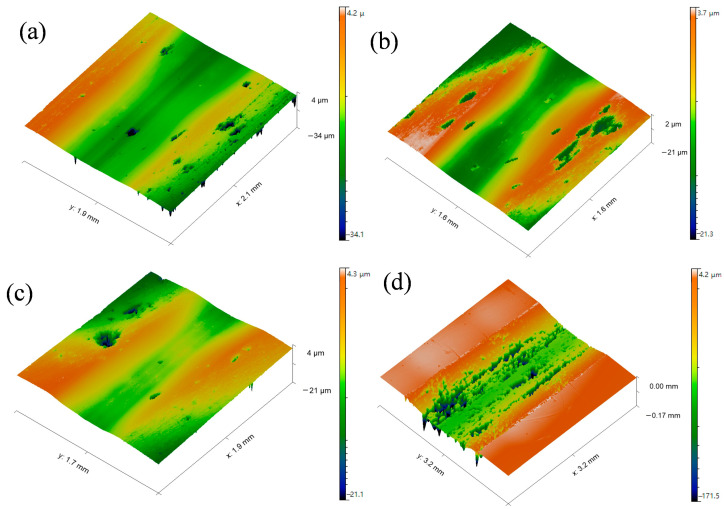
Three-dimensional morphology of local wear scars on WC-CoCrNiAlTi coating samples and the substrate: (**a**) AFR-1.078; (**b**) AFR-1.104; (**c**) AFR-1.130; (**d**) 04Cr13Ni5Mo.

Figure 6 displays the three-dimensional morphology of the wear tracks on the WC-CoCrNiAlTi coatings and the 04Cr13Ni5Mo substrate after dry sliding wear. It can be observed that the width and depth of the wear tracks for all coatings are significantly smaller than those of the 04Cr13Ni5Mo stainless steel substrate. Furthermore, the cross-sectional profiles of the wear tracks (Figure 5c) indicate that both the depth and width of the wear tracks on the WC-CoCrNiAlTi coatings gradually decrease with increasing AFR. Correspondingly, the wear rate of the coatings also gradually decreases with rising AFR (Figure 5d). At an AFR of 1.130, the coating exhibits the lowest wear rate of 1.15 × 10^−6^ mm^3^·N^−1^·m^−1^, demonstrating wear resistance that is 34.85 times higher than that of the 04Cr13Ni5Mo martensitic stainless-steel substrate. Combined with the hardness results shown in Figure 4, it is evident that the wear resistance of the WC-CoCrNiAlTi coatings is closely related to their hardness. According to classical wear theory, hardness is one of the key factors influencing the wear resistance of materials [27]. It is generally accepted that the wear rate of a material is inversely proportional to its hardness, meaning that higher hardness corresponds to better wear resistance. However, the tribological and wear performance of the HVAF-sprayed WC-CoCrNiAlT coating is inferior to that of the reported HVAF-sprayed WC-10Co4Cr coating (with a wear rate of about 5.0 × 10^−8^ mm^3^·N^−1^·m^−1^) [15]. This may be attributed to the formation of the hard and brittle (Co, Ni)_3_W_3_C phase, which is prone to cracking under frictional loading, leading to the initiation of microcracks.

**Figure 7:**
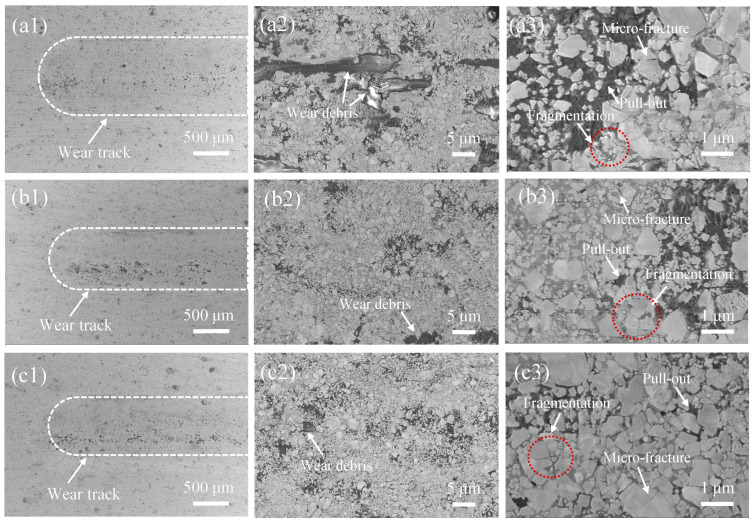
SEM morphology of the worn surfaces of HVAF-sprayed WC-CoCrNiAlTi coating samples: (**a1**–**a3**) AFR-1.078; (**b1**–**b3**) AFR-1.104; (**c1**–**c3**) AFR-1.130.

Figure 7 shows the SEM morphology of the worn surfaces of different coating samples after friction and wear testing. From Figure 7(a1–c1), it can be seen that the width of the wear track gradually decreases with increasing AFR, indicating a reduction in material loss. This result is consistent with the trend in wear rate shown in Figure 5. In Figure 7(a2), a large amount of wear debris and localized plowing grooves can be observed on the worn surface of the AFR-1.078 coating. This is primarily attributed to the spalling of hard and brittle phases within the coating during friction, forming abrasive particles that enter the friction interface. These particles cause micro-plowing and micro-cutting actions on the coating surface, thereby intensifying abrasive wear. At higher magnification, the wear track (Figure 7(a3)) reveals typical damage features on the surfaces of some carbide particles, including localized microcracks, fragmentation, and pull-out detachment, particularly in high-porosity regions where the bonding phase provides weaker local support. Localized areas within the wear track are filled with dark wear debris, which often corresponds to pre-existing pore defects in the original coating microstructure. These pores tend to act as vulnerable sites for the damage of WC particles. During the wear process, these loose and weak areas are typically worn away or spalled off preferentially. Once the support from the binder phase is weakened, the embedded WC particles become unstable, easily loosened, pulled out, or entirely dislodged [28]. In contrast, the worn surface of the AFR-1.130 coating, prepared at a higher AFR, is much smoother with a uniform distribution of WC particles and no significant wear debris (Figure 7(b1)). This is because this coating possesses higher density, hardness, and toughness. Its synergistic strong-yet-tough structure effectively resists friction and wear, resulting in excellent wear resistance. Therefore, the sliding wear behavior of the WC-CoCrNiAlTi coating results from the synergistic interaction between the wear of the ductile binder (micro-plowing/micro-cutting) and the damage of hard carbides (fracture/pull-out).

### 3.4. Erosion Wear Resistance

**Figure 8:**
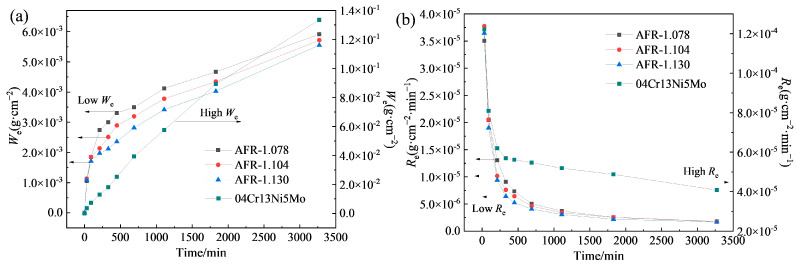
Slurry erosion resistance of HVAF-sprayed WC-CoCrNiAlTi coatings: (**a**) erosion weight loss curves; (**b**) erosion rate curves.

Figure 8a presents the cumulative weight loss curves of the WC-CoCrNiAlTi coatings and the 04Cr13Ni5Mo stainless steel substrate under slurry erosion conditions. It can be observed that the weight loss per unit area of all materials gradually increases with prolonged erosion time. After 210 min of erosion, coatings prepared at different AFRs exhibit significant differences in erosion resistance: a higher AFR corresponds to a lower erosion weight loss, indicating superior resistance to slurry erosion wear. At the highest AFR of 1.130, the coating demonstrates the lowest weight loss of merely 5.55 × 10^−3^ g·cm^−2^ representing an erosion resistance 24.04 times that of the stainless-steel substrate. As seen from the erosion rate curves in Figure 8b, the coating samples show a relatively high erosion rate during the initial stage, which gradually decreases and stabilizes after approximately 1800 min, entering a steady-state erosion stage. In contrast, the erosion rate of the 04Cr13Ni5Mo stainless steel continues to decline over time. Among the coatings fabricated at the three different AFRs, the AFR-1.130 coating exhibits the lowest steady-state erosion rate of 1.70 × 10^−6^ g·cm^−2^·min^−1^, which is only 4.12% of that measured for the 04Cr13Ni5Mo stainless steel. The performance surpasses the erosion wear resistance of HVAF-sprayed WC-10Co4Cr coatings and HVOF-sprayed WC-10Co4Cr coatings reported in the literature, which are approximately 1.0 × 10^−6^ kg/m^2^/s (6 × 10^−6^ g·cm^−2^·min^−1^) and 2.0 × 10^−6^ kg/m^2^/s (1.2 × 10^−5^ g·cm^−2^·min^−1^) [15].

**Figure 9:**
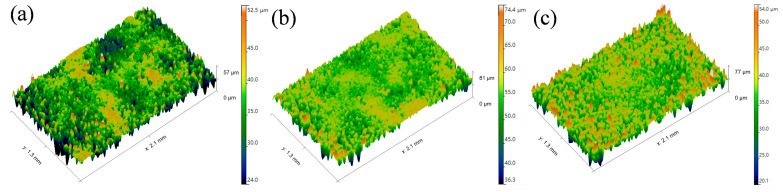
3D morphology of the wear scars on HVAF-sprayed WC-CoCrNiAlTi coating samples: (**a**) AFR-1.078; (**b**) AFR-1.104; (**c**) AFR-1.130.

Figure 9 presents the 3D surface morphology of the WC-CoCrNiAlTi coating after slurry erosion wear. It can be observed that under low-AFR conditions, relatively large and deep erosion pits appear on the coating surface. As the AFR increases, the depth of the erosion pits on the coating surface decreases and the surface gradually becomes flatter. Statistical results of the surface roughness reveals that the average Sa for the AFR-1.078, AFR-1.104, and AFR-1.130 coatings are 6.115 μm, 5.448 μm, and 4.613 μm, respectively. The gradual decrease in Sa with increasing AFR reflects an improvement in slurry erosion resistance.

**Figure 10:**
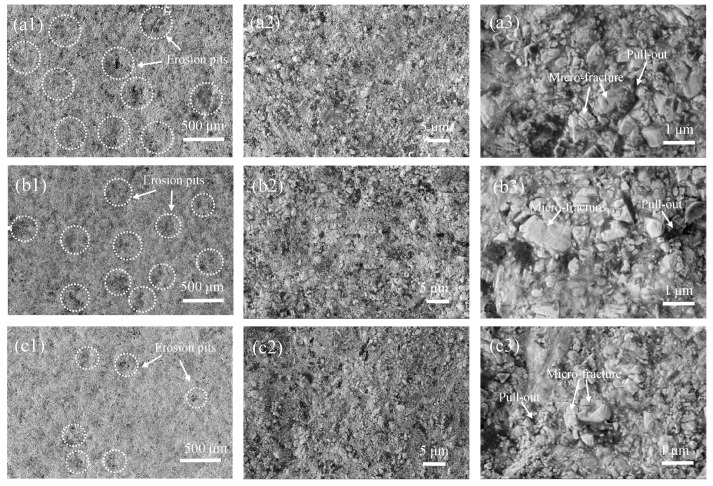
Surface morphology of the wear scars on HVAF-sprayed WC-CoCrNiAlTi coating samples: (**a1**–**a3**) AFR-1.078; (**b1**–**b3**) AFR-1.104; (**c1**–**c3**) AFR-1.130.

To further analyze the erosion wear mechanism of the WC-CoCrNiAlTi coating, the surface micro-morphology after 54.5 h of slurry erosion was examined using SEM. As shown in Figure 10(a1), uneven erosion pits are observed on the eroded coating surface. With increasing AFR, the size and number of erosion pits on the coating surface gradually decrease, which is consistent with the observations in Figure 9. Higher-magnification images reveal localized wear on the coating surface, with densely exposed carbide particles (Figure 10(a2)). This phenomenon is attributed to the preferential removal of the softer binder phase during erosion, which exposes the embedded WC hard particles, forming “micro-protrusions” that act as an anti-wear skeleton. Simultaneously, the ductile HEA binder phase provides effective support and protection to the brittle WC phase through adaptive plastic deformation, thereby delaying overall coating damage.

Further observation at higher magnification (Figure 10(a3)) reveals residual pits left by particle spallation, along with fatigue microcracks identified on some carbide surfaces. This is caused by the accumulation of fatigue stress in WC particles under continuous erosive load, leading to cleavage fracture or particle fragmentation, which results in localized spallation of the coating under the combined action of sand particle impact, slurry penetration, and hydraulic forces [29]. Thus, the density of the coating and the interfacial bonding strength between the WC particles and the binder phase play critical roles in determining its slurry erosion resistance. Although the wear morphology and failure mechanisms are generally similar for coatings prepared at different AFRs, notable differences exist: coatings produced at a low AFR, characterized by higher porosity and weaker bonding between WC particles and the matrix, are more susceptible to wear initiation at interfacial regions. In contrast, the coating prepared at a high AFR exhibits higher density and stronger interfacial bonding, resulting in better resistance to fatigue spallation under alternating erosive stress.

## 4. Conclusions

WC-based cermet coatings with CoCrNiAlTi binder were fabricated on 04Cr13Ni5Mo stainless steel substrates via atmospheric high-velocity air–fuel (HVAF) spraying. The influence of the air-to-fuel ratio (AFR = 1.078, 1.104, and 1.130) on the microstructure, mechanical properties, and wear resistance of the WC-CoCrNiAlTi coatings was systematically investigated. The friction wear and erosion mechanisms of the composite coatings were elucidated. The main conclusions are as follows:

(1) All WC-CoCrNiAlTi coatings consist primarily of WC, (Co, Ni)_3_W_3_C, and an FCC binder phase. As the AFR increases, the formation of the (Co, Ni)_3_W_3_C phase gradually decreases. Meanwhile, the coating density improves, which is attributed to enhanced particle melting and higher impact velocity, resulting in improved flattening upon deposition.
(2) The average microhardness of the WC-CoCrNiAlTi coatings gradually increases with increasing AFR. The coating sprayed at an AFR of 1.130 exhibits the highest microhardness of 1355.68 HV_0.2_. This is due to the combined effects of reduced hard and brittle (Co, Ni)_3_W_3_C decomposition phases and improved microstructural densification.
(3) Both the friction coefficient and the wear rate of the coatings decrease with increasing AFR. At an AFR of 1.130, the coating demonstrates the lowest friction coefficient (0.6435) and wear rate (1.15 × 10^−6^ mm^3^·N^−1^·m^−1^). Its wear resistance is 34.85 times higher than that of the 04Cr13Ni5Mo martensitic stainless-steel substrate.
(4) With prolonged slurry erosion time, the cumulative weight loss of the WC-CoCrNiAlTi coatings increases, while the erosion rate decreases. As the AFR increases, the weight loss rate of the coatings gradually declines. The coating produced at an AFR of 1.130 shows the lowest erosion rate (1.70 × 10^−6^ g·cm^−2^·min^−1^), exhibiting slurry erosion resistance 24.04 times greater than that of the 04Cr13Ni5Mo stainless steel substrate.
(5) The slurry erosion mechanism of the WC-CoCrNiAlTi coatings is attributed to the fatigue-induced removal of WC particles under prolonged erosive impact. The AFR significantly influences the slurry erosion resistance by regulating the content of brittle decomposition phases and the density of the coatings.

## Figures and Tables

**Table 1:** Chemical composition of the HVAF-sprayed WC-CoCrNiAlTi coatings (at.%).

Samples	Region	C	W	Co	Cr	Ni	Al	Ti
Nominal	WC	50	50	-	-	-	-	-
	binder	-	-	60	25	5	5	5
AFR-1.078	WC	50.05	46.14	2.36	1.18	0.22	0.03	0.02
	binder	-	29.13	45.87	18.26	3.30	1.17	2.27
AFR-1.104	WC	49.72	45.62	2.65	0.97	0.67	0.13	0.24
	binder	-	24.76	47.35	20.94	3.51	1.23	2.21
AFR-1.130	WC	49.91	45.94	2.58	1.17	0.33	0.03	0.04
	binder	-	22.20	51.54	19.03	3.65	1.30	2.28

## Data Availability

The original contributions presented in this study are included in the article. Further inquiries can be directed to the corresponding authors.
